# Overview of biotherapy in rheumatoid arthritis (RA)

**DOI:** 10.1186/ar3596

**Published:** 2012-02-09

**Authors:** Tsutomu Takeuchi, Hideto Kameda

**Affiliations:** 1 Division of Rheumatology, Department of Internal Medicine, School of Medicine, Keio University, Shinjyuku,Tokyo, Japan

## 

Biological agents targeting a specific molecule provide an effective means for therapeutic management of rheumatoid arthritis (RA) due to their specificity and powerful functional capabilities, which has resulted in a paradigm shift in the treatment strategy of this disease. The dramatic improvement of the sign and symptoms of a patient with RA first came from the report with chimeric anti-TNF alpha monoclonal, infliximab in 1993. The observation was confirmed in the double-blind randomized controlled study comparing this biological agent and placebo in 1994. The first approved biologics for RA was TNF Receptor 1-Ig fusion protein, etanercept in the United States in 1998. Until now, nine biological agents are approved in RA worldwide. Revolutionary change of RA management with biological therapies obtained in western countries and Japan has been reviewed [1].

Atreatment strategy that uses tightly controlled dosesof administered biologics, targeting clinical remission or low disease activity, and followed by discontinuation of the biologics may be advantageous from botha health and economical point of view. This strategy is now being examinedin several clinical studies and trials in Japan for several biologics, including infliximab, etanercept, tocilizumab, and abatacept [1].

It is ideal to personalize medical treatment for individual RA patients by predicting efficacy and safety of a given biologic. In order to identify predictive factors, enormous amounts of efforts have put forth. Although several clinical variables have been associated with efficacy and safety, they are often unrealistic in clinical practice. We found that the baseline circulating TNF levels [2] and Fc gamma 3B polymorphism [3] are important predicting factors for response to infliximab in RA patients, and discuss the role of these markers in real world. Further clinical studies using biomarkers and molecular expression pattern [4,5] should provide a clue to find the appropriate predicting markers or even new therapeutic targets. In the near future, the information accumulated from these studies may allow selecting the best biological agents in individual patient.
